# The Prognostic Value of the Acute Phase Systemic Immune–Inflammation Index in Patients With Intracerebral Hemorrhage

**DOI:** 10.3389/fneur.2021.628557

**Published:** 2021-05-25

**Authors:** Yunke Li, Dingke Wen, Wenyao Cui, Yuqi Chen, Fazhen Zhang, Maolin Yuan, Han Xiao, Hao Li, Lu Ma, Xin Hu, Chao You

**Affiliations:** ^1^Neurosurgery Department of West China Hospital, Sichuan University, Chengdu, China; ^2^Medical School of Sichuan University, Chengdu, China

**Keywords:** stroke, prognosis, blood platelets, intracerebral hemorhage, neutrophils lymphocyte ratio

## Abstract

**Background and Purpose:** The systemic immune–inflammation index (SII) is a novel prognostic index in various diseases. We evaluated the predictive value of SII in patients with intracerebral hemorrhage (ICH).

**Methods:** Patients with primary spontaneous ICH were enrolled. SII was constructed based on peripheral platelet (P), neutrophil (N), and lymphocyte (L) and defined as P^*^N/L. In addition to admission testing, acute phase SII was collected to analyze the potential dynamic change. Poor outcome was defined as modified Rankin Scale of more than 3 at 90 days.

**Results:** We included 291 patients; 98 (34%) achieved favorable functional outcomes. Day-1 SII was higher and was more related to poor outcome than was admission SII. Median time of day-1 SII was 29 h from onset. Day-1 SII had an OR in outcome (mRS >3) 1.74 (95% CI = 1.03–3.00, *p* = 0.04). The binary cutoff point of SII calculated using the area under the curve (AUC) method was 1,700 × 10^9^/L, AUC 0.699 (95% CI = 0.627–0.774) (sensitivity 53.3%, specificity 77.3%) (OR = 2.36, 95% CI = 1.09–5.26, *p* = 0.03).

**Conclusions:** SII, especially day-1 SII, was highly associated with 90-day functional outcome in patients with ICH and could be used to predict outcomes.

## Introduction

Spontaneous intracerebral hemorrhage (ICH) is associated with high mortality and poor outcome (1). Studies found various indicators for predicting outcome following ICH (2–4); however, few of these involve biochemical tests. The brain injury after ICH includes the primary injury, which is the mechanical damage of the adjacent tissues by hematoma within the first hours after ICH, and the secondary injury, which is initiated by the extravasation of blood products into the brain parenchyma (5). Mounting preclinical evidence has shown that inflammation after ICH plays an important role in the secondary brain injury (6). Furthermore, clinical laboratory results that reflect inflammation have been reported to predict ICH outcome. Platelet (PLT) counts have been associated with growth edema (7), which predicts ICH outcome (8). The neutrophil–lymphocyte ratio (NLR) was reported (9, 10), as were PLT–lymphocyte ratio (PLR) (11), and lymphocyte–monocyte ratio (LMR) (12). These studies showed that levels of inflammation are highly related to the clinical outcome following ICH (13).

The systemic immune-inflammation index (SII), which was calculated as peripheral platelet^*^neutrophil/lymphocyte, was first reported as a prediction tool in cancers such as hepatocellular carcinoma (14–16). In 5,602 coronary artery disease patients after coronary intervention, SII was shown to have a better prediction for major cardiovascular events than traditional risk factor (17). In acute ischemic stroke, SII was reported as an independent risk factor for stroke severity (18). Moreover, dynamic changes of SII was suggested as a promising prognostic predictor for cancer patients such as colorectal cancer and hepatocellular carcinoma (19, 20). A recent study confirmed the value of SII for predicting short-term outcome following ICH (21). Nevertheless, the role of SII in predicting long-term outcome following ICH is unknown.

In the present study, we investigated acute phase SII and favorable outcome of ICH in recovery. We also studied dynamic changes of SII to identify a more precise way to predict outcome.

## Methods

The data and code that support the findings of this study are available from the corresponding author upon reasonable request. The study was approved by the local ethic committee.

We retrospectively collected data from patients admitted to West China Hospital, Sichuan University (Sichuan, China) from February 2018 to February 2019. The inclusion criteria were as follows: (1) over 18 years of age; (2) admission diagnosis of ICH based on brain CT scans; (3) <24 h from onset to admission; (4) available clinical data including at least one laboratory test of platelets, neutrophils, lymphocytes and monocytes; and (5) neuro-image to evaluate the characteristics of the hematoma. Exclusion criteria were as follows: (1) secondary ICH (aneurysm, vascular malformation, or tumor); (2) possible disease that may affect laboratory results (leukemia, lymphoma, or thrombocytopenia); and (3) unavailability of outcome data; (4) patients with coagulopathy or anticoagulant therapy; and (5) patients with active infection or autoimmune disease.

We recorded age, sex, clinical record, previous medical history, laboratory results (PLT), absolute neutrophil count (ANC), absolute lymphocyte count (ALC), absolute monocyte count (AMC), baseline CT imaging characteristics, and surgical information if available. ICH volume was measured based on the ABC/2 method (22). We collected all laboratory test results during hospitalization. SII was defined as platelet^*^neutrophil/lymphocyte.

The primary outcome was modified Rankin scale (mRS) at 90 days from onset. mRS was measured at outpatient visit or by telephone using a structured interview (23). We defined favorable outcome as mRS 0–3, and unfavorable outcome was mRS 4–6.

All statistical analyses were performed using R software (Version 4.0.2, R Core Team, Vienna, Austria). Continuous variables were tested using the Student's *t* test or Mann–Whitney test and were expressed as mean (standard deviation) or median (interquartile range) depending on their distribution. Categorical variables were defined as numbers and were analyzed using the χ^2^ test or Fisher exact test. Receiver operating curves (ROCs) were generated and the area under curve (AUC) was calculated to estimate the ability of the SII and other factors to predict poor outcomes. The optimal cutoff point was calculated using the Youden's Index. Boxplots were performed to describe the distribution of admission and day-1 SII in ordinal mRS. Multivariate logistic regression analysis was used to analyze the association between factors and prognosis. Variables included in the model were selected based on the result of univariate analysis. Two-tailed *p* < 0.05 was considered significant.

## Results

We enrolled 291 patients (Supplementary Figure 1). Of these, 98 (34%) achieved favorable outcomes at 90 days. The poor outcome group included more females (38 vs. 23%, *p* = 0.02), older patients (59 ± 14 vs. 55 ± 13, *p* = 0.0002), lower Glasgow Coma Scales [8 (6–13) vs. 13 (13–15), *p* < 0.0001], larger ICH volumes [32 (14–59) vs. 12 (4–24), *p* < 0.0001], and more intraventricular hematomas (64 vs. 27%, *p* < 0.001) (Table 1). Admission ANC and day-1 ANC were significantly higher in patients with unfavorable outcome, and they tended to have a lower ALC on day-1. Admission SII and day-1 SII both showed significant differences between outcome groups (Table 1 and Supplementary Table 1). The interval of the day-1 test in the full cohort was 29 (13–51) h from the onset; no difference was found between both groups.

**Table 1:** Baseline comparison between 90-day outcome groups.

	**Full cohort (*n* = 291)**	**Favorable outcome (*n* = 98)**	**Poor outcome (*n* = 193)**	***P-*value**
Age, years	57 (14)	55 (13)	59 (14)	0.0002^*^
Male sex	194 (67%)	75 (77%)	119 (62%)	0.02^†^
Onset to admission time, h	5 (3–8)	6 (3–10)	5 (3–8)	0.20^‡^
Admission SBP, mmHg	166 (143–183)	163 (144–181)	166 (142–184)	0.71^‡^
Admission DBP, mmHg	94 (82–109)	95 (82–111)	93 (81–108)	0.41^‡^
Admission GCS	13 (7–14)	13 (13–15)	8 (6–13)	<0.0001^‡^
Admission SII, × 10^9^/L	1298 (658–2244)	989 (570–1867)	1440 (792–2422)	0.004^‡^
Day−1 SII, × 10^9^/L	1467 (884–2485)	969 (685–1564)	1833 (1170–2955)	<0.0001^‡^
Craniotomy	63 (22%)	12 (12%)	51 (26%)	0.008^†^
ICH volume, ml	24 (9–47)	12 (4–24)	32 (14–59)	<0.0001^‡^
Intraventricular hematoma	145 (50%)	22 (27%)	123 (64%)	<0.0001^†^
Lobar hematoma	52 (18%)	20 (12%)	32 (17%)	0.52^†^
Infratentorial hematoma	58 (20%)	16 (16%)	42 (22%)	0.35^†^

*Data are mean (standard deviation) or median (interquartile range) for continuous variables, and n (%) for categorical variables. SBP, systolic blood pressure; DBP, diastolic blood pressure; GCS, glasgow coma scale; SII, systemic immune–inflammation index; ICH, intracerebral hemorrhage*.

* *χ2 test or Fisher exact test*.

† *Two-sample Student's t test*.

‡ *ann–Whitney test*.

The multivariate analysis was carried out considering factors including SII, sex, age, ICH volume (logarithm), IVH extension, hematoma location, and craniotomy. In multivariate analysis, day-1 SII independently predicted 90-day poor outcome (OR 1.74, 95% CI = 1.03–3.00, *p* = 0.04), while admission SII did not (OR 1.19, 95% 0.81–1.75, *p* = 0.37) (Table 2). Receiver operating characteristics yielded a cutoff of 1,315 × 10^9^/L for admission SII (AUC 0.726, sensitivity 83.7%, specificity 56.9%) and 1,700 × 10^9^/L for day-1 SII (AUC 0.699, sensitivity 53.3%, specificity 77.3%) with corresponding maximum Youden index for predicting 90-day outcome (Supplementary Figure 2). Multivariate analysis revealed that Day1-SII > 1,700 × 10^9^/L (OR 2.36, 95% CI = 1.09–5.26, *p* = 0.03), but not admission SII > 1,315 × 10^9^/L (OR 1.42, 95% CI = 0.72–2.82, *p* = 0.31), was significantly associated with poor 90-day functional outcome (Supplementary Table 2). Moreover, age, GCS, ICH volume, and location were also found as an independent predictor [detailed in(Supplementary Table 2)].

**Table 2:** Relationship of SII and SII threshold with 90-day predicting poor outcome.

	**Unadjusted**			Adjusted^*^		
	**OR**	**95% CI**	***P*-value**	**OR**	**95% CI**	***P*-value**
Admission SII^†^	1.47	1.12–1.96	0.007	1.19	0.81–1.75	0.37
Day-1 SII^†^	2.87	1.91–4.54	<0.0001	1.74	1.03–3.00	0.04
Admission SII >1315 × 10^9^/L	2.14	1.31–3.55	0.003	1.42	0.72–2.82	0.31
Day-1 SII >1700 × 10^9^/L	4.51	1.21–4.44	<0.0001	2.36	1.09–5.26	0.03

*SII, systemic immune–inflammation index*;

* *Adjusted by sex, age, admission Glasgow Coma Scale, logarithm intracerebral hematoma volume, intraventricular hematoma occurrence, hematoma location and craniotomy*;

† *Logarithm*.

**Figure 1:**
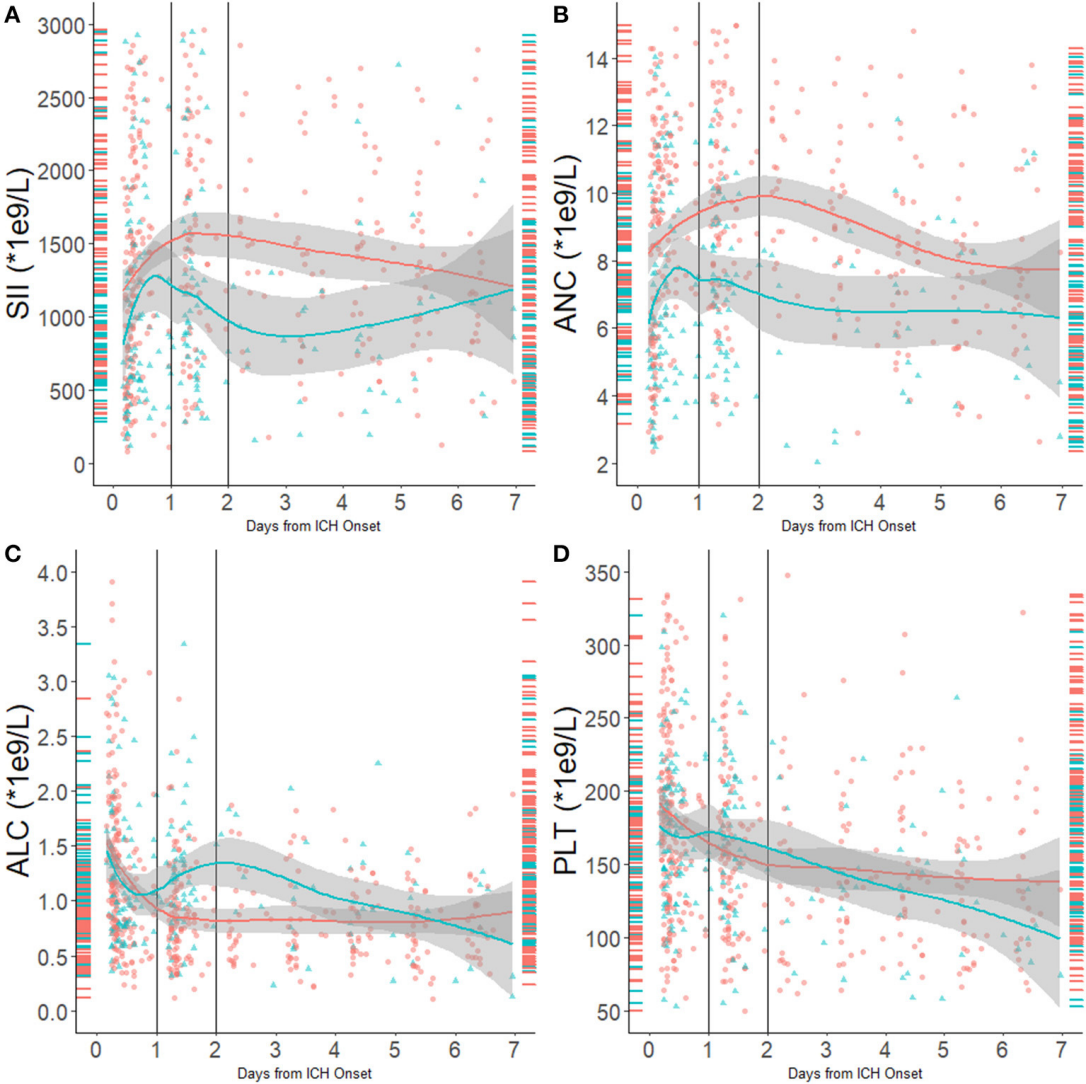
**(A)** SII distribution and fitting curve according to 90-day outcome; **(B)** ANC distribution and fitting curve according to 90-day outcome; **(C)** ALC distribution and fitting curve according to 90-day outcome; **(D)** PLT distribution and fitting curve according to 90-day outcome. Red indicates patients with unfavorable outcomes, and blue indicates patients with favorable outcomes. Right-side rungs indicate SII distribution in the first 12 h from onset; the left side indicates SII distribution 24–48 h from onset.

**Figure 2:**
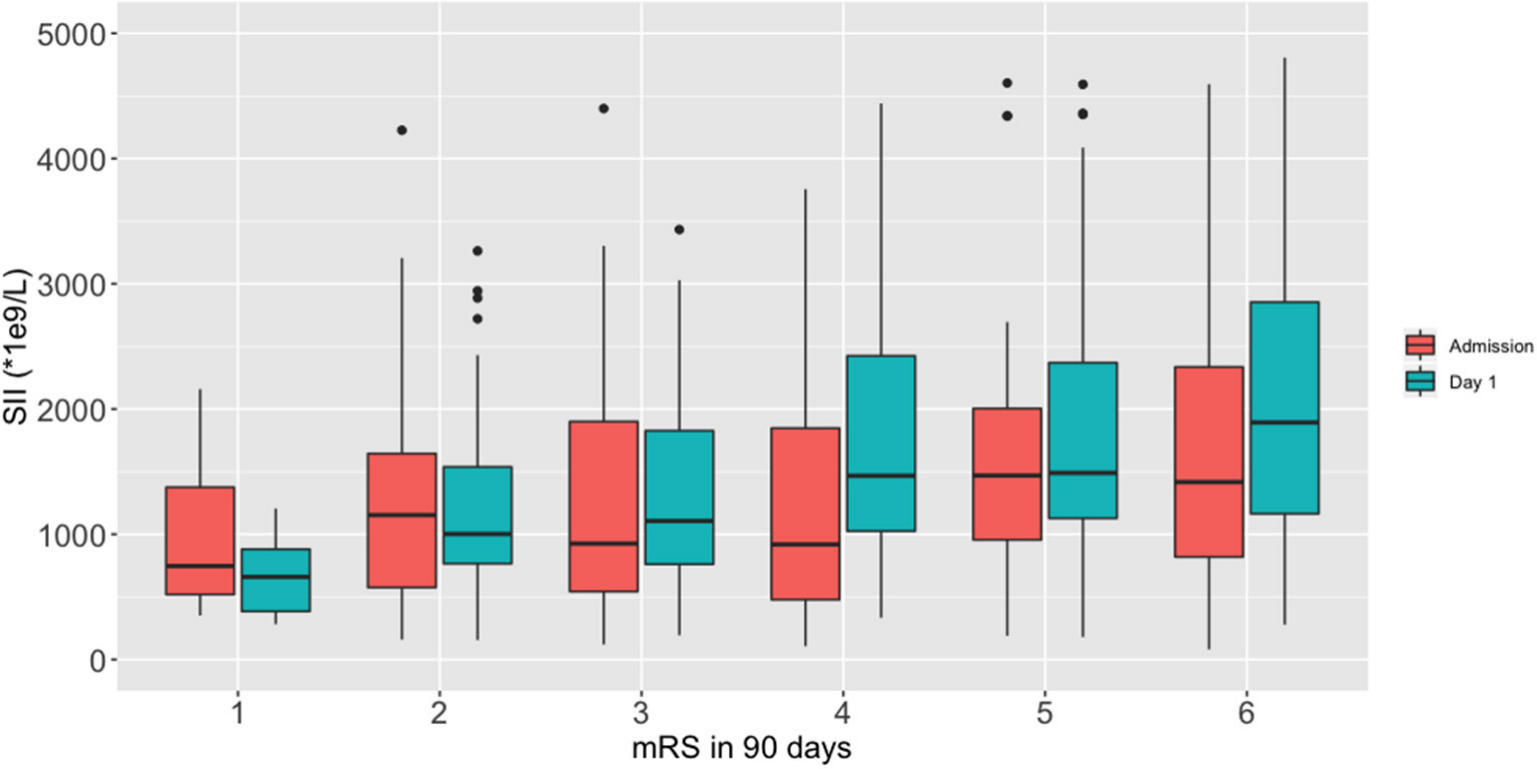
Boxplots of admission and day-1 SII stratified by ordinal mRS.

We performed a fitting curve based on the binary functional outcome and their individual ANC, ALC, PLT, and SII to display the trends (Figure 1). A peak of SII occurred at 24–48 h after stroke, and an obvious gap was identified after 24 h from ICH onset. ANC showed the same trend as SII, and ALC showed an inverse curve. PLT did not show a significant difference on the fitting curve. Furthermore, boxplots of admission and day-1 SII stratified by ordinal mRS revealed different distribution of SII, especially day-1 SII, in each mRS category (Figure 2).

## Discussion

We described the dynamic change of the SII in ICH patients and detected a peak at 24–48 h after ICH onset. The admission SII and day-1 SII was found to be associated with 90-day poor outcome. In the multivariate analysis, only day-1 SII independently predicted 90-day functional outcome with an optimal cutoff at 1,700 × 10^9^/L. These findings suggest that the SII might serve as a new important indicator for prognosis prediction and risk stratification in ICH patients. To our knowledge, this was the first study that reported the dynamic change of SII following ICH and evaluated the predicting value of SII in long-term functional outcome in ICH patients.

There is accumulating evidence that inflammatory indices calculated based on routine blood count such as neutrophils and lymphocytes can provide valuable prognostic information in various diseases including ICH and ischemic stroke (9, 24–26). Since these inflammatory indices are easily obtainable and widely accessible, they could be added as simple predictive tools for risk stratification during clinical estimation. Meanwhile, the increase of these inflammatory indices might also reflect the acute inflammatory response to the primary and secondary brain injury.

After ICH occurs, plasma-derived factors (i.e., thrombin and vitronectin) and components released following erythrocyte lysis (i.e., hemoglobin, peroxiredoxin 2, and carbonic anhydrase 1) can activate macrophage/microglia and trigger the inflammatory cascade (27, 28). Activated macrophages/microglia further release pro-inflammatory cytokines and chemokines and promote infiltration of peripheral inflammatory cells (29). Neutrophils are the earliest white blood cells recruited from peripheral blood to the brain in response to acute inflammatory immune response (10). In animal models, neutrophil infiltration was observed around the hematoma within 4 h and reached a peak in 1–3 days after ICH (6, 30). By analyzing the tissues surrounding the hematoma in patients with ICH, there was neutrophil infiltration within 8 h that further increased within 1 day (31). Neutrophils induce neurotoxicity by releasing pro-inflammatory cytokines (i.e., TNF-α and IL-1β), further contributing to increased capillary permeability, blood–brain barrier destruction, and aggravation of brain edema (32). In preclinical research, targeting neutrophil inhibition alleviated myelin fragmentation and axonal damage, further improving functional outcomes after ICH (33).

Platelets are an integral component of the hemostatic system (28). The balance of platelet aggregation is broken after ICH. The increase of platelet counts in the peripheral circulation induces a hypercoagulable state, which increases the risk of poor outcomes (34). Activated platelets release a series of potent chemical mediators (i.e., adenosine diphosphate, serotonin, thromboxane A2, and TGF β), all of which may potentially play important roles in brain damage and unfavorable prognosis (28).

In the acute phase after ICH, the sympathetic system and hypothalamic–pituitary–adrenal axis are overactivated and the levels of catecholamines and steroids increase, which contribute to systemic immunosuppression and further induce functional inactivation and apoptosis of peripheral lymphocytes (35). Lymphocytes play a crucial role in immune regulation and host defense against pathogens (10). Decreases in lymphocyte numbers reduces the immune capacity, increases the risk of infection after ICH, and may have an impact on functional outcomes (34, 36).

Based on previous studies, the inflammatory immune response may not be reflected in laboratory tests within the first few hours of onset (31). In our cohort, the median onset to admission time was 5 h, which is substantially shorter than that of a previous report (21); as a result, the immune response may not fully reach the ultra-acute phase, giving rise to a different result. A more precise observation of inflammatory immune markers in stroke patients may be needed in the future.

There are some limitations in our study. A single-center retrospective cohort has potential biases. Other inflammatory markers such as edema volume and interleukins were not collected, and the interaction with other infective complications were not studied. The strengths of our report include the dynamic change of the immune–inflammation index. A previous study focused on the admission time point but did not consider the actual time from onset of stroke (21). We also had a relatively wide enrollment of patients with ICH, including all locations of hematoma and surgical patients.

## Conclusion

SII is an easily calculated index that showed decent ability to predict outcome following ICH. Further investigations may increase the understanding of immune–inflammation processes in ICH and may guide clinical practice.

## Data Availability Statement

The data and code that support the findings of this study are available from the corresponding author upon reasonable request.

## Ethics Statement

The studies involving human participants were reviewed and approved by Medical ethics committee of Sichuan University. Written informed consent for participation was not required for this study in accordance with the national legislation and the institutional requirements.

## Author Contributions

YL and XH designed the study. YL, YC, FZ, MY, and HX collected the data. YL and DW analyzed the data. DW, WC, and XH did major revision. All authors contributed to the article and approved the submitted version.

## Conflict of Interest

The authors declare that the research was conducted in the absence of any commercial or financial relationships that could be construed as a potential conflict of interest.
