# Supplementary material for: The Prognostic Value of the Acute Phase Systemic Immune–Inflammation Index in Patients With Intracerebral Hemorrhage
Source: Front Neurol. 2021 May 25;12:628557. doi: 10.3389/fneur.2021.628557 (PMC8185273; doi:10.3389/fneur.2021.628557)
Supplement: Supplementary file 1 [file Data_Sheet_1.PDF]

SUPPLEMENTAL MATERIAL

P2. Figure I. Flow chart of patient selection

P3. Table I. Baseline Comparison between 90-day outcome groups

P4-P5. Figure II. Adjusted receiver operating curve and area under curve of SII

a. Admission SII——P4
b. Day-1 SII——P5

P6. Table II. Multivariate logistic regression models predicting 90-day poor outcome

Figure I. Flow chart of patient selection

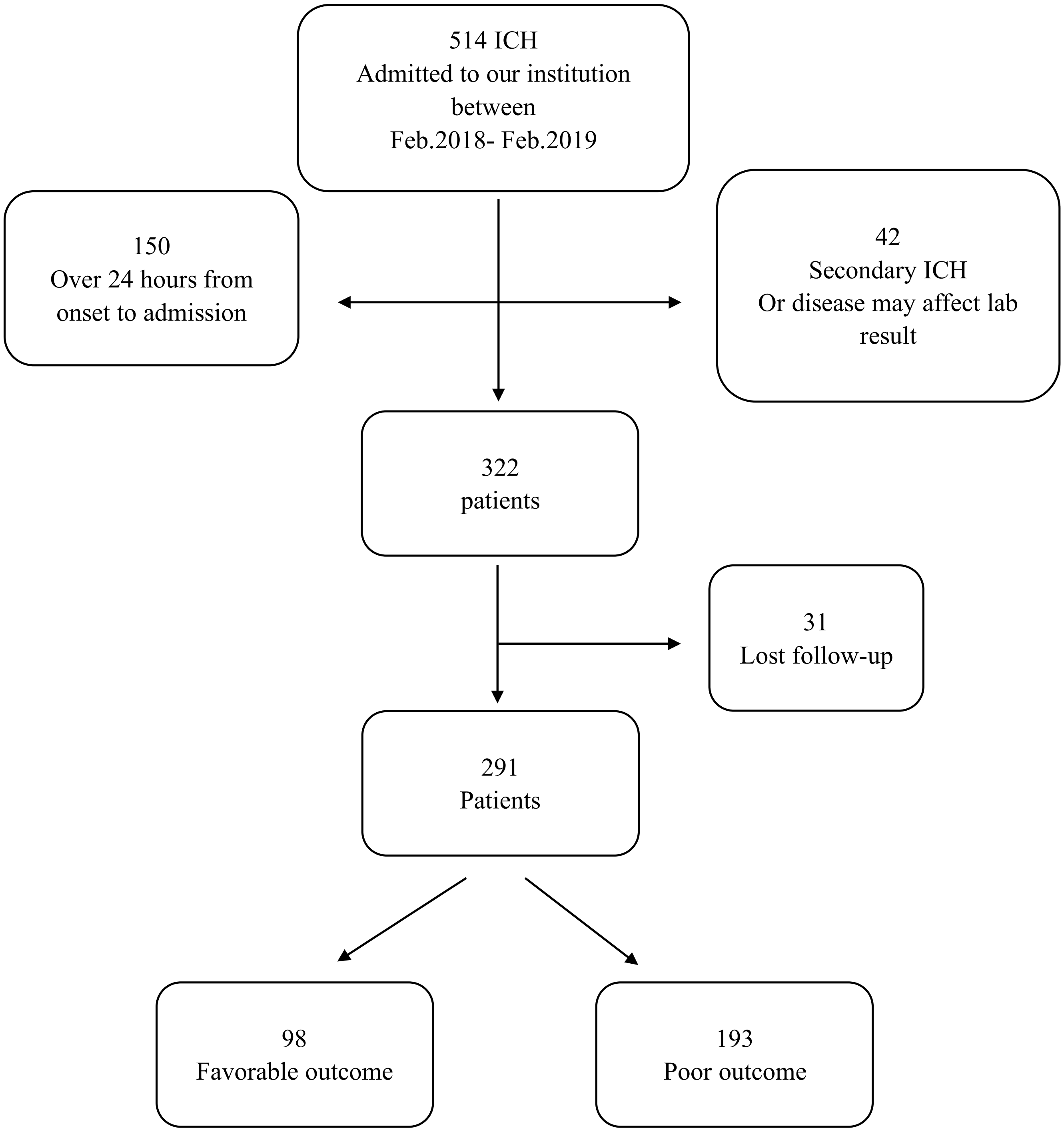

Table I. Baseline Comparison between 90-day outcome groups

| | Full cohort (n=291) | Favorable Outcome (n=98) | Poor outcome (n=193) | *P* value |
|---|---|---|---|---|
| Hypertension | 180 (62%) | 57 (58%) | 123 (64%) | 0.43* |
| Diabetes mellitus | 28 (9%) | 10 (10%) | 18 (9%) | 0.97* |
| Chronic kidney disease | 8 (3%) | 2 (2%) | 6 (3%) | 0.72* |
| Coronary artery disease | 7 (2%) | 1 (1%) | 6 (3%) | 0.43* |
| Anticoagulated | 9 (3%) | 2 (2%) | 7 (4%) | 0.72* |
| Antiplatelets | 22 (8%) | 8 (8%) | 14 (8%) | 0.95* |
| Prior stroke | 22 (8%) | 8 (8%) | 14 (8%) | 0.97* |
| Current smoker | 97 (33%) | 37 (38%) | 60 (31%) | 0.31* |
| Alcohol abuse | 37 (13%) | 16 (16%) | 21 (11%) | 0.25* |
| Admission PLT, *$10^9$/L | 176 [130-215] | 172 [137-203] | 180 [128-233] | 0.29† |
| Day-1 PLT, *$10^9$/L | 155 [118-200] | 160 [127-200] | 151 [108-199] | 0.35† |
| Admission ANC, *$10^9$/L | 9.2 [5.7-11.7] | 6.4 [4.8-10.0] | 10.0 [6.9-12.5] | <0.0001† |
| Day-1 ANC, *$10^9$/L | 8.8 [6.7-11.5] | 7.3 [5.5-9.0] | 9.9 [7.4-12.9] | <0.0001† |
| Admission ALC, *$10^9$/L | 1.12 [0.76-1.65] | 1.03 [0.78-1.54] | 1.14 [0.76-1.77] | 0.43† |
| Day-1 ALC, *$10^9$/L | 0.90 [0.63-1.29] | 1.16 [0.82-1.49] | 0.80 [0.53-1.11] | <0.0001† |

PLT, platelets; ANC, absolute neutrophil count; ALC, absolute lymphocyte count

*$\chi2$ test or Fisher exact test

†Mann-Whitney test

Figure II Unadjusted receiver operating curve and area under curve of SII

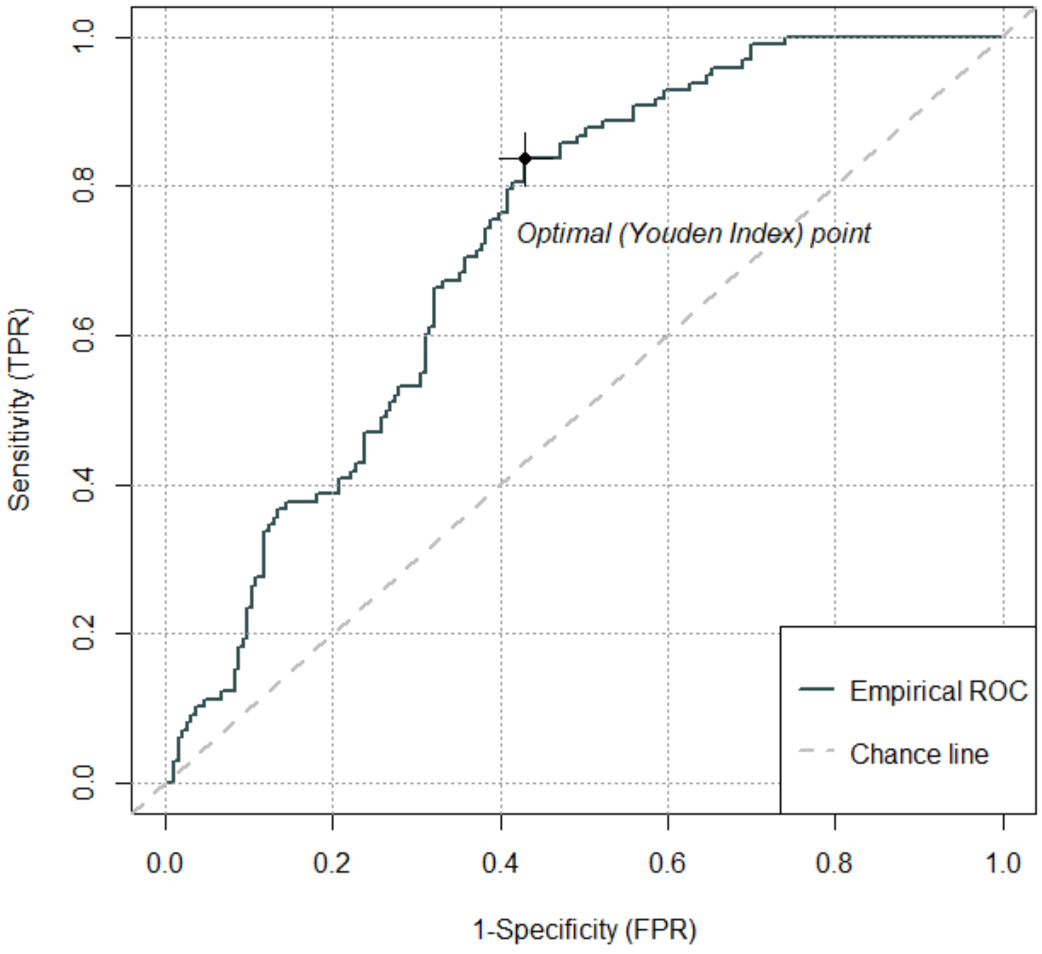


a. Admission SII

Area under curve 0.726 (95%CI 0.662-0.791)
Optimal point (Admission SII=1315*$10^9$/L) sensitivity 83.7%, specificity 56.9%.

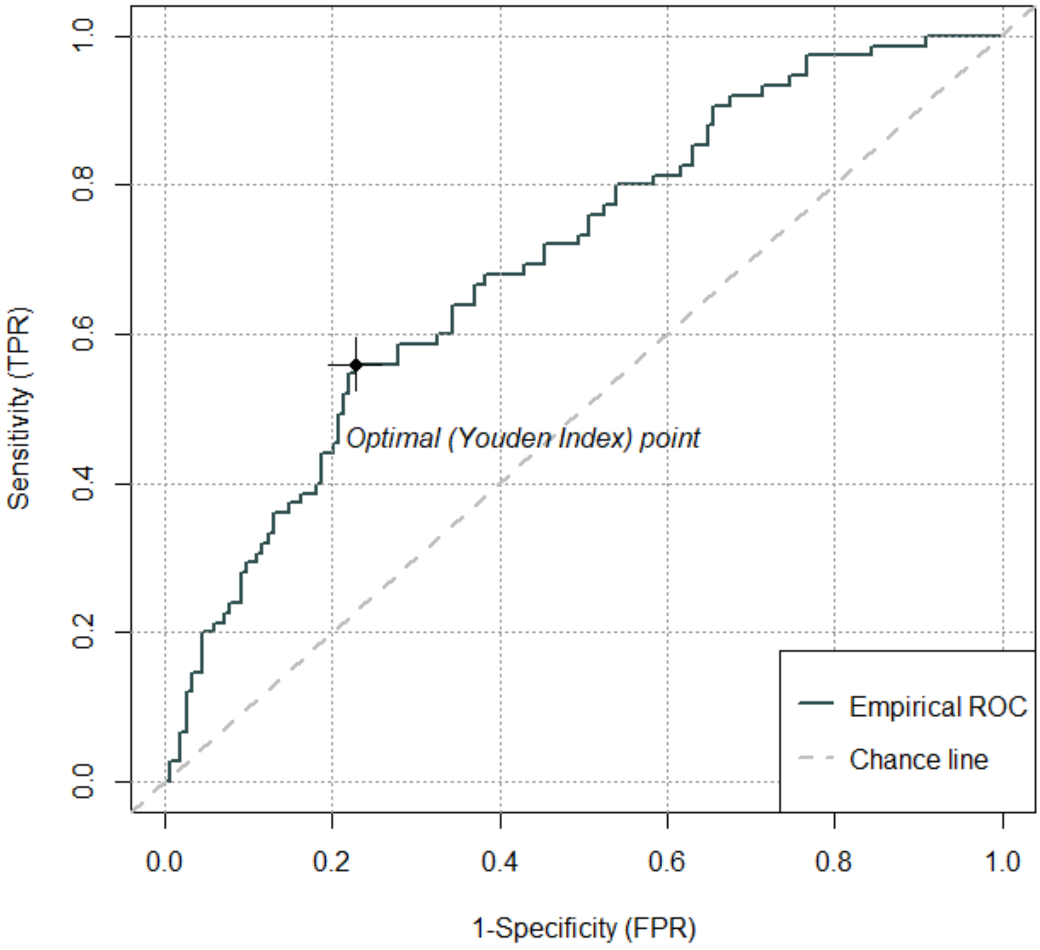


b. Day-1 SII

Area under curve 0.699 (95%CI 0.624-0.774)
Optimal point (Day-1 SII=1700*$10^9$/L), sensitivity 53.3%, specificity 77.3%.

Table II. Multivariate logistic regression models predicting 90-day poor outcome

| | Odds Ratio | 95% CI lower | 95% CI upper | *P* Value |
|---|---|---|---|---|
| **Model 1** | | | | |
| Male | 0.53 | 0.25 | 1.09 | 0.09 |
| Age | 1.03 | 1.01 | 1.06 | 0.007 |
| GCS | 0.73 | 0.64 | 0.82 | <0.0001 |
| *Log* ICH volume | 2.18 | 1.41 | 3.52 | 0.0008 |
| IVH | 1.86 | 0.91 | 3.85 | 0.09 |
| ICH Location | 2.18 | 1.08 | 4.56 | 0.03 |
| Craniotomy | 1.19 | 0.48 | 3.05 | 0.71 |
| Admission SII | 1.19 | 0.81 | 1.75 | 0.37 |
| **Model 2** | | | | |
| Male | 0.62 | 0.28 | 1.67 | 0.24 |
| Age | 1.04 | 1.01 | 1.07 | 0.004 |
| GCS | 0.79 | 0.69 | 0.90 | 0.0005 |
| *Log* ICH volume | 2.31 | 1.39 | 4.07 | 0.002 |
| IVH | 1.67 | 0.77 | 3.64 | 0.19 |
| ICH Location | 2.86 | 1.28 | 6.60 | 0.01 |
| Craniotomy | 0.97 | 0.37 | 2.85 | 0.95 |
| Day-1 SII | 1.74 | 1.03 | 3.00 | 0.04 |
| **Model 3** | | | | |
| Male | 0.53 | 0.25 | 1.07 | 0.08 |
| Age | 1.03 | 1.01 | 1.06 | 0.006 |
| GCS | 0.73 | 0.64 | 0.82 | <0.0001 |
| *Log* ICH volume | 2.18 | 1.42 | 3.52 | 0.0007 |
| IVH | 1.82 | 0.88 | 3.76 | 0.10 |
| ICH Location | 2.18 | 1.08 | 4.52 | 0.03 |
| Craniotomy | 1.19 | 0.48 | 3.06 | 0.71 |
| Admission SII>1315 $*10^9/L$ | 1.42 | 0.72 | 2.82 | 0.31 |
| **Model 4** | | | | |
| Male | 0.63 | 0.28 | 1.38 | 0.26 |
| Age | 1.04 | 1.01 | 1.06 | 0.007 |
| GCS | 0.79 | 0.68 | 0.89 | 0.0003 |
| *Log* ICH volume | 2.38 | 1.44 | 4.17 | 0.001 |
| IVH | 1.70 | 0.79 | 3.72 | 0.17 |
| ICH Location | 2.90 | 1.31 | 6.70 | 0.01 |
| Craniotomy | 0.97 | 0.37 | 2.59 | 0.95 |
| Day1-SII>1700 $*10^9/L$ | 2.36 | 1.09 | 5.26 | 0.03 |

Note: variables included in the multivariate logistic regression:

model 1: Male, age, GCS, ICH volume, IVH, ICH location, craniotomy, Admission SII
model 2: Male, age, GCS, ICH volume, IVH, ICH location, craniotomy, Day-1 SII
model 3: Male, age, GCS, ICH volume, IVH, ICH location, craniotomy, Admission SII>1315 $*10^9$/L
model 4: Male, age, GCS, ICH volume, IVH, ICH location, craniotomy, Day1-SII>1700 $*10^9$/L
